# A Randomized Comparison of Dihydroartemisinin-Piperaquine and Artesunate-Amodiaquine Combined With Primaquine for Radical Treatment of Vivax Malaria in Sumatera, Indonesia

**DOI:** 10.1093/infdis/jit407

**Published:** 2013-08-06

**Authors:** Ayodhia Pitaloka Pasaribu, Watcharee Chokejindachai, Chukiat Sirivichayakul, Naowarat Tanomsing, Irwin Chavez, Emiliana Tjitra, Syahril Pasaribu, Mallika Imwong, Nicholas J. White, Arjen M. Dondorp

**Affiliations:** 1 Faculty of Tropical Medicine, Mahidol University, Bangkok, Thailand; 2 Medical Faculty, University of Sumatera Utara, Medan, North Sumatera, Indonesia; 3 Center for Emerging and Neglected Infectious Diseases, Mahidol University, Bangkok, Thailand; 4 National Institute of Health Research and Development, Ministry of Health, Jakarta, Indonesia; 5 Centre for Tropical Medicine, Nuffield Department of Medicine, University of Oxford, United Kingdom

**Keywords:** primaquine, radical cure, *Plasmodium vivax*, Indonesia

## Abstract

***Background.*** A high prevalence of chloroquine-resistant *Plasmodium vivax* in Indonesia has shifted first-line treatment to artemisinin-based combination therapies, combined with primaquine (PQ) for radical cure. Which combination is most effective and safe remains to be established.

***Methods.*** We conducted a prospective open-label randomized comparison of 14 days of PQ (0.25 mg base/kg) plus either artesunate-amodiaquine (AAQ + PQ) or dihydroartemisinin-piperaquine (DHP + PQ) for the treatment of uncomplicated monoinfection *P. vivax* malaria in North Sumatera, Indonesia. Patients were randomized and treatments were given without prior testing for G6PD status. The primary outcome was parasitological failure at day 42. Patients were followed up to 1 year.

***Results.*** Between December 2010 and April 2012, 331 patients were included. After treatment with AAQ + PQ, recurrent infection occurred in 0 of 167 patients within 42 days and in 15 of 130 (11.5%; 95% confidence interval [CI], 6.6%–18.3%) within a year. With DHP + PQ, this was 1 of 164 (0.6%; 95% CI, 0.01%–3.4%) and 13 of 143 (9.1%; 95% CI, 4.9%–15.0%), respectively (*P* > .2). Intravascular hemolysis occurred in 5 patients, of which 3 males were hemizygous for the G6PD-Mahidol mutation. Minor adverse events were more frequent with AAQ + PQ.

***Conclusions.*** In North Sumatera, Indonesia, AAQ and DHP, both combined with PQ, were effective for blood-stage parasite clearance of uncomplicated *P. vivax* malaria. Both treatments were safe, but DHP + PQ was better tolerated.

***Clinical Trials Registration.*** NCT01288820.

Approximately 2.6 billion people are at risk of acquiring *Plasmodium vivax* infection worldwide, of whom half live in Southeast Asia [1]. In contrast with *Plasmodium falciparum* malaria, *P. vivax* can cause relapse infections emerging from dormant hypnozoite forms in the liver. Strains in tropical regions such as Sumatera are characterized by frequent (>30%) and early (around 1 month) relapses [2]. Radical cure can only be achieved by adding a hypnozoitocidal drug, and the 8-aminoquinolone primaquine (PQ) is the only widely available drug for this purpose [3]. However, the drug is used infrequently because of concerns about its oxidative side effects causing intravascular hemolysis and methemoglobinemia in populations in whom glucose-6-phosphate dehydrogenase (G6PD) deficiency is common and facilities for assessing G6PD status are not readily available (ie, most malaria-endemic areas). The G6PD gene is located on the X chromosome and there are >180 genetic polymorphisms, most of which confer reductions in G6PD-enzyme activity [4]. The common variants differ importantly in their effect on enzyme activity; hence, the associated risk of hemolysis after PQ treatment varies enormously. The prevalence of G6PD deficiency is approximately 5% in North Sumatra [5], but which variants are prevalent and the risks vs benefits of deploying PQ are not known.

*Plasmodium vivax* resistance to chloroquine is prominent in many parts of Indonesia, ranging from 43% in Sumatera island to >80% in Papua [6–8], In 2008, artesunate-amodiaquine (AAQ) and, more recently, dihydroartemisinin-piperaquine (DHP) have replaced chloroquine as first-line treatments [9, 10]. However, it has not been established which of these artemisinin combination therapies (ACTs) is most effective in Sumatera. We compared the efficacy and safety of AAQ + PQ and DHP + PQ for the treatment of uncomplicated vivax malaria in the operationally realistic context without prior testing for G6PD deficiency to identify the optimal treatment of vivax malaria.

## MATERIALS AND METHODS

We performed a prospective, open-label, randomized study comparing AAQ + PQ and DHP + PQ for the treatment of uncomplicated symptomatic *P. vivax* monoinfection in nonpregnant adults and children aged >1 year presenting at a rural clinic in Tanjung Leidong village, Labuhan Batu, North Sumatera, Indonesia. Routine G6PD testing is not available here. Clinical malaria incidence is 400–500 per year among a population of 32 837 (in 2010), equally divided between *P. vivax* and *P. falciparum* infections (written communication, July 2011, from Ministry of Health, Indonesia).

Patients with fever (or recent fever <48 hours) and microscopically confirmed *P. vivax* monoinfection (≥250/µL) were eligible. Exclusion criteria included any feature of severe malaria [3], severe malnutrition, recurrent vomiting, concomitant infections, pregnancy or lactation, known allergies to the study medication, and inability to follow up. Written informed consent was obtained from patients or their attending relatives before enrollment.

The study was approved by the Ethics Committee of the National Institute of Health Research and Development, Indonesian Ministry of Health, Jakarta, Indonesia; Faculty of Tropical Medicine, Mahidol University, Thailand; and the Oxford Tropical Research Ethics Committee, Oxford University, United Kingdom.

Parasite density was assessed per 200 white blood cells on a Giemsa-stained thick film, and assumed to be absent if not detected in 200 high-power fields. Gametocytes were counted per 1000 white blood cells. Parasite species was confirmed in thin smear, and 10% of slides were cross-checked at the Faculty of Tropical Medicine, Mahidol University. Other investigations included hemoglobin measurement (Hemocue201+), hemoglobin-methemoglobinemia by pulse oximetry (Masimo-Set, Masimo), and G6PD genotyping from a filter paper blood spot (Whatman 3M). Genotyping by polymerase chain reaction–restriction fragment-length polymorphism (PCR-RFLP) enabled identification of 3 common mutations (Mediterranean, Mahidol, and Viangchan) [11]. In patients developing hemolysis or methemoglobinemia with no mutation by PCR-RFLP, and in patients identified as G6PD deficient by a fluorescent spot test at the end of the study (see below), sequencing of the whole G6PD gene was performed (Macrogen).

Patients were not screened for G6PD status before the start of therapy and were managed as outpatients, both current practice in Sumatera. All patients were followed daily for 14 days and then weekly until 42 days, followed by monthly visits up to a year, or in between in case of symptoms. Hemoglobin levels were assessed on days 0, 2, and 7, and then weekly. During PQ therapy, methemoglobinemia was monitored daily. PQ therapy was discontinued in case of macroscopic hemoglobinuria, a drop in hemoglobin >2 g/dL, or when methemoglobin increased to >20% of total hemoglobin. At the end of the study, all patients were invited to test for G6PD status using a NADPH qualitative spot test (SQMMR720 kit, R&D Diagnostics).

Patients randomized to AAQ (Arsuamoon, Guilin Pharmaceuticals) received artesunate 12 mg/kg and amodiaquine 30 mg/kg divided over 3 days. Patients randomized to DHP (Arterakine, Pharbaco Central Pharmaceuticals), received dihydroartemisinin 6.75 mg/kg and piperaquine 54 mg/kg in divided doses over 3 days. All patients also received PQ (Phapros Inc) in a dose of 0.25 mg base/kg (or 15 mg for >40 kg) for 14 days started on the first day. All treatment doses were given directly observed and together with some biscuits (ie, cookies). If the patient vomited within 30 minutes, the dose was repeated. Recurrent vivax malaria infections occurring in the first 42 days of follow-up were treated with quinine/doxycycline following Indonesian guidelines; episodes occurring after this point were treated with the same regimen as the initial treatment. All patients were provided with insecticide-treated bednets.

Patients were randomized by an independent statistician in blocks of 10, with each treatment allocation concealed in an opaque, sealed envelope, opened only after enrollment.

### Outcome

Patient outcomes, including early treatment failure, late treatment failure, and adequate clinical and parasitological response, were classified according to World Health Organization guidelines [12]. The primary outcome was 42-day efficacy. Secondary outcomes included risk of recurrent *P. vivax* infection during 1-year follow-up, fever and parasitemia clearance times, gametocyte carriage rates and clearance times, hematological recovery, and safety and tolerability of treatments.

### Statistical Analysis

Including a 10% anticipated loss, a sample size of 165 patients per study arm was calculated to detect a difference in 42-day cure rate of 90% with AAQ + PQ vs 98% with DHP + PQ with 95% confidence and 80% power.

Data were anonymized and double entered into a secured database (OpenClinica). Analysis was done using Stata software (StataCorp). The primary intention-to-treat analysis included all randomized patients and per-protocol analysis of all patients who completed 42 days of follow-up. Comparisons between groups were made by Mann–Whitney *U* test, Student *t* test, χ^2^ test, and Fisher exact test where appropriate. Efficacy at 42 days and after 1 year of follow-up were assessed by Kaplan–Meier survival analysis with log-rank test for statistical significance.

## RESULTS

**Figure 1.**
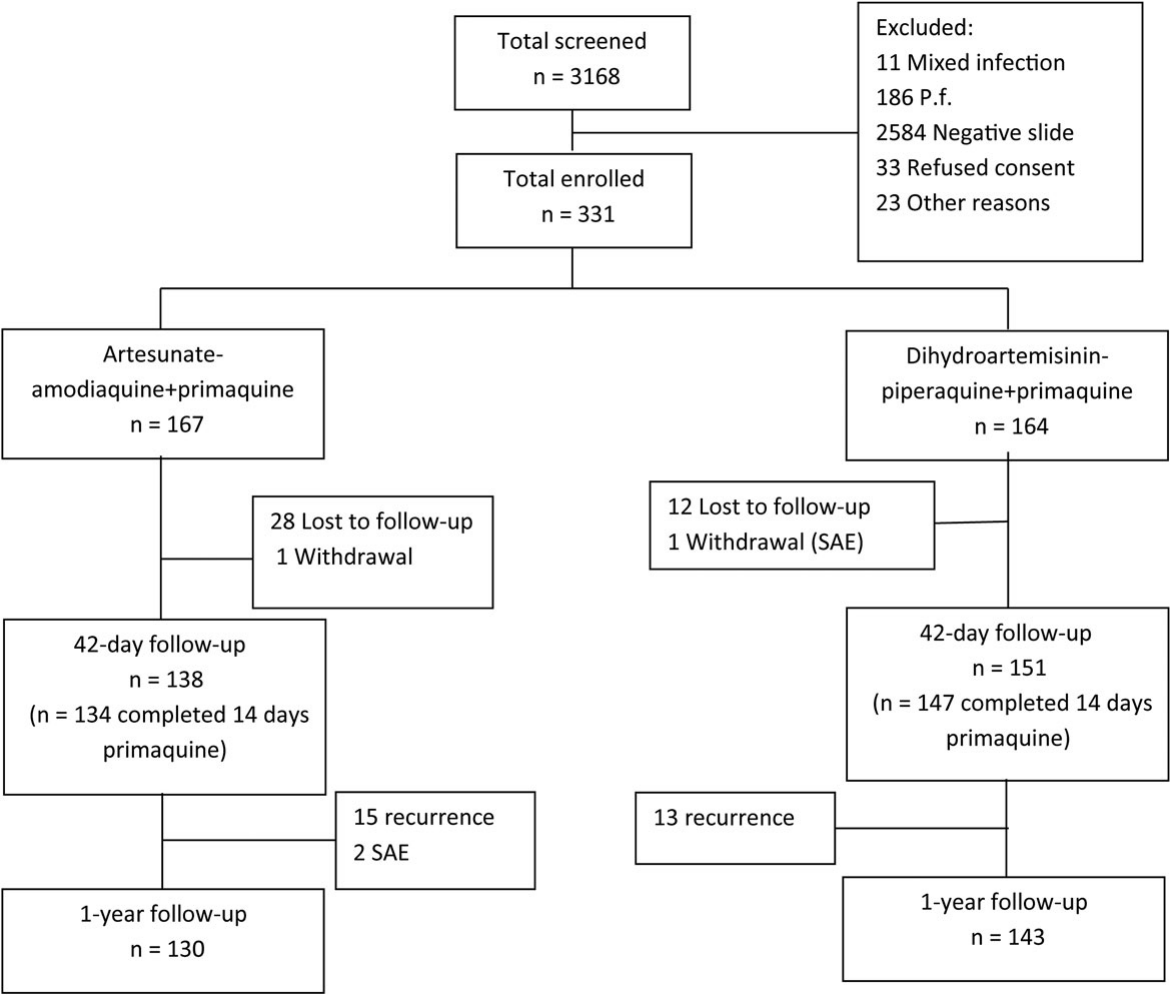
Study flowchart. Abbreviations: *P.f.*, *Plasmodium falciparum*; SAE, severe adverse event.

Between December 2010 and April 2012, 3168 patients were screened, of whom 331 were enrolled in the study. A total of 167 patients were treated with AAQ + PQ and 164 with DHP + PQ (Figure 1). Baseline characteristics were similar between treatment arms (Table 1). Follow-up until day 42 was achieved for 138 of 167 (83%) patients treated with AAQ + PQ and 151 of 164 (91%) with DHP + PQ. One-year follow-up was completed in 130 of 167 (78%) patients treated with AAQ + PQ and 143 of 164 (87%) with DHP + PQ. The median number of missed visits per patient completing 1 year of follow-up was 1 (range, 0–9) for both treatment arms.

**Table 1.** Patient Characteristics at Baseline

Characteristic	AAQ + PQ (n = 167)	DHP + PQ (n = 164)
Geometric mean of asexual *Plasmodium vivax*/µL (95% CI)	1061 (876–1285)	981 (811–1187)
Patients with gametocytes on admission	67 (40.1)	74 (45.1)
Sex		
Female	66 (39.5)	79 (48.2)
Male	101 (60.5)	85 (51.8)
Weight, kg, median (range)	38 (9–99)	37 (10–80)
Age, y, median (range)	13 (2–63)	14.5 (2–70)
Age group		
<18 y	106 (64.2)	96 (59.3)
≥18 y	59 (35.8)	66 (40.8)
Temperature, mean (SD)	37.7 (1.0)	37.7 (1.0)
≥37.5°C, No. (%)	92 (55.1)	96 (58.5)
<37.5°C, No. (%)	75 (44.9)	68 (41.5)
Hemoglobin concentration (mean, SD)	12 (1.5)	11.7 (1.4)
≥10 g/dL, No. (%)	151 (90.4)	148 (90.2)
<10 g/dL, No. (%)	16 (9.6)	16 (9.8)
Methemoglobin concentration, mean (SD)	1.63 (0.82)	1.59 (0.95)
Repellent use	38 (29.7)	39 (32.7)
Insecticide-treated net use	96 (60.4)	105 (67.7)
History of antimalarial use	28 (20)	22 (15.9)
Occupation		
Unemployed	20 (12.1)	19 (11.8)
Fisherman	56 (33.9)	52 (32.3)
Laborer	27 (16.4)	31 (19.2)
Housewife	8 (4.9)	7 (4.3)
Businessman	9 (5.4)	6 (3.7)
Teacher	4 (2.4)	4 (2.5)
Student	26 (15.8)	26 (16.1)
Policeman	3 (1.8)	3 (1.8)
Farmer	12 (7.3)	13 (8.1)
Education		
Primary	1 (0.8)	3 (2.4)
Junior high	70 (53.4)	61 (48.4)
Senior high	27 (20.6)	29 (23.0)
University	29 (22.1)	23 (18.2)
No education	4 (3.1)	10 (7.9)

Data are presented as No. (%) unless otherwise indicated.

Abbreviations: AAQ, artesunate-amodiaquine; CI, confidence interval; DHP, dihydroartemisinin-piperaquine; PQ, primaquine.

### Therapeutic Response

**Figure 2.**
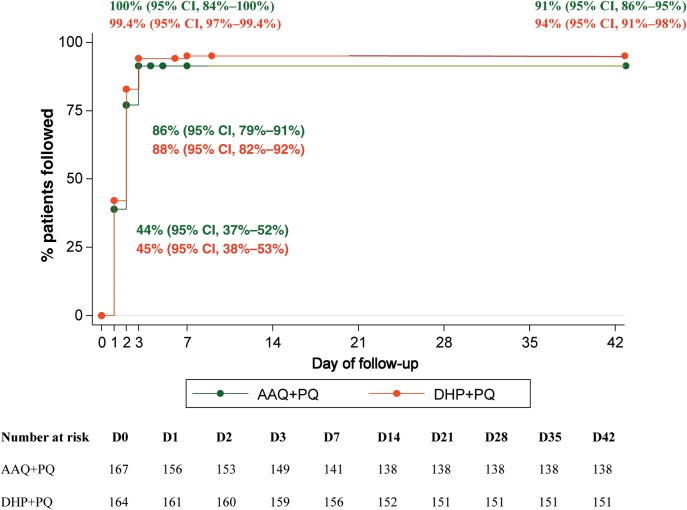
Kaplan–Meier survival efficacy analysis of all randomized patients. Abbreviations: AAQ + PQ, artesunate-amodiaquine plus primaquine; CI, confidence interval; DHP + PQ, dihydroartemisinin-piperaquine plus primaquine.

**Figure 3.**
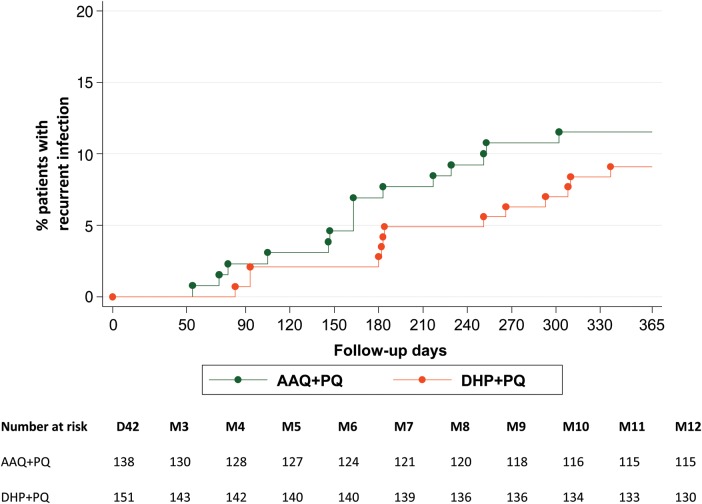
Kaplan–Meier analysis for recurrent infection during the 1-year follow-up period. Abbreviations: AAQ + PQ, artesunate-amodiaquine plus primaquine; DHP + PQ, dihydroartemisinin-piperaquine plus primaquine.

Intention-to-treat survival analysis showed an adequate parasitological cure rate at 42 days of 91% (95% confidence interval [CI], 86%–95%) with AAQ + PQ and 94% (95% CI, 91%–98%) with DHP + PQ (Figure 2, log-rank *P* = .51). Per-protocol analysis of patients with complete 42-day follow-up showed cure rates of 100% (95% CI, 98%–100%; 138 of 138 patients) with AAQ + PQ and 99.3% (95% CI, 97%–99.9%; 150 of 151 patients) with DHP + PQ (*P* = .31). Parasite clearance was within 48 hours in both treatment arms, except for 1 patient with early treatment failure after DHP + PQ (who received rescue treatment) and another 2 patients after DHP + PQ who cleared parasites after >72 hours; neither showed recurrent infection during follow-up. No late treatment failures until day 42 were found in either treatment group. During 1-year follow-up, recurrent infections were observed in 15 of 130 (11.5%) patients after AAQ + PQ (of whom 2 had a second recurrent *P. vivax* infection) and 13 of 143 (9.1%) after DHP + PQ (Figure 3, log-rank *P* = .48). The earliest recurrence after treatment with AAQ + PQ was at day 54 compared to 83 days after DHP + PQ. After 1 year, the mean day of recurrence was day 165 (SD, 70) for patients treated with AAQ + PQ and day 203 (SD, 91) for those treated with DHP + PQ (*P* = .23). Among 28 patients with recurrent infections, 24 had monoinfection with *P. vivax*, 2 had monoinfection with *P. falciparum*, and 2 had mixed infection (*P. falciparum/P. vivax*). Cumulative risk of recurrence for the total group during the 1-year follow-up period was 17.5 per 100 person-years.

On admission, 92 of 167 (55.1%) patients in the AAQ + PQ arm and 96 of 164 (58.5%) in the DHP + PQ arm had fever (≥37.5°C). All patients treated with DHP + PQ cleared their fever within 1 day, compared to 89 of 92 (97%) with AAQ + PQ (*P* = .07). In patients presenting with gametocytemia, 55 of 67 (82%) of patients treated with AAQ + PQ and 63 of 74 (85%) with DHP + PQ cleared gametocytemia within day 1 (*P* = .63), and all patients cleared gametocytemia by day 2. At day 42, the mean hemoglobin was 11.9 g/dL (95% CI, 11.8–12.1 g/dL) with DHP + PQ vs 11.9 g/dL (95% CI, 11.7–12.1 g/dL) with AAQ + PQ (*P* = .91). Hemoglobin levels did not differ between treatment arms at any time point.

### Adverse Events

In patients treated with AAQ + PQ, 3 had a drop in hemoglobin level >2 g/dL (to 7.9 g/dL, 12.3 g/dL, and 10.9 g/dL, respectively), of whom 2 developed cola-colored urine temporarily without other complications. One patient had an increased methemoglobin level of 20.3%, after which PQ was discontinued. One patient developed a generalized urticarial rash half an hour after the first dose of AAQ + PQ. This patient recovered after treatment with an antihistamine and was subsequently treated with quinine/doxycycline. In patients treated with DHP + PQ, 1 male and 1 female patient had a drop in hemoglobin level >2 g/dL (to 8.8 g/dL and 7.8 g/dL, respectively) and 2 had increased methemoglobin levels to 20.2% and 21.6% respectively, after which PQ was discontinued. None of the patients with intravascular hemolysis needed blood transfusion, and hemoglobin levels returned to normal (>10 g/dL) after a median of 14 (range, 7–35) days. An increase of >10% in methemoglobin level occurred in 17 of 167 (10.2%) patients treated with AAQ + PQ compared to 24 of 164 (14.6%) treated with DHP + PQ (*P* = .22).

All 8 patients with PQ-related hemolysis or methemoglobinemia were genotyped. Three male patients with hemolysis were hemizygous for the Mahidol variant of the G6PD gene. One male and 1 female patient with hemolysis had normal results on both PCR-RFLP and complete gene sequencing. The 3 patients with methemoglobinemia also had normal results on PCR-RFLP. Another 52 patients without hemolysis or methemoglobinemia were genotyped. All had the normal reference genotype, except for 1 female patient who was heterozygous for the Mahidol variant.

At the end of the study, 212 of 273 (78%) patients were screened for G6PD status by fluorescence spot test. Two males and 5 females (2.6%) were G6PD deficient according to the screening test. The median reduction in hemoglobin levels in these patients was 1.4 g/dL (range, 0.9–2 g/dL). Gene sequencing showed that 1 male patient was hemizygous for the Mahidol variant and another male carried the 1311C→T intron 11 nt93T→C mutation. One of the 5 females was heterozygous for the C 1311 T/C intron 11 nt 93 T/C and intron 2 nt 8 C/A mutations, whereas the other 4 had wild-type genotype (Table 2).

**Table 2.** Summary of G6PD Status Analysis

Patient No.	Sex	Symptom	Hb Drop, g/dL	FST	Genotyping	Sequencing
1	M	Dark urine/Hb drop	10.9 to 7.9	−	Mahidol	−
2	M	Dark urine/Hb drop	14.9 to 12.3	+	Mahidol	−
3	M	Hb drop	13.7 to 10.9	−	Normal	Normal
4	M	Hb drop	12.7 to 8.8	−	Mahidol	−
5	F	Hb drop	10.5 to 7.8	−	Normal	Normal
6	F	MetHb rise		Normal	Normal	−
7	F	MetHb rise		Normal	Normal	−
8	M	MetHb rise		Normal	Normal	−
9	F	−		+	Mahidol (heterozygous)	−
10	M	−		+	−	Mahidol
11	M	−		+	−	1311 C→T intron 11 nt 93 T→C
12	F	−		+	−	Normal
13	F	−		+	−	Normal
14	F	−		+	−	Normal
15	F	−		+	−	Normal
16	F	−		+	−	C 1311 T/C intron 11 nt 93 T/C and intron 2 nt 8 C/A (heterozygous)

Abbreviations: FST, f^l^uorescent spot test; Hb, hemoglobin; MetHb, methemoglobin.

Minor adverse events were more commonly reported in patients receiving AAQ + PQ compared to those receiving DHP + PQ (Table 3).

**Table 3.** Adverse Events

Adverse Event	AAQ + PQ (n = 167),No. (%)	DHP + PQ (n = 164), No. (%)	*P* Value
Headache	92 (55.1)	50 (30.5)	.001
Dizziness	24 (14.4)	7 (4.4)	.002
Vomiting	86 (51.5)	8 (4.9)	<.001
Diarrhea	27 (16.2)	8 (4.9)	.08
Skin rash	4 (2.4)	1 (0.6)	.37
Dyspnea	6 (3.6)	0 (0.0)	.03
Abdominal pain	46 (27.5)	14 (8.5)	.001
Hemolysis	3 (1.8)	2 (1.2)	>.50

Abbreviations: AAQ, artesunate-amodiaquine; DHP, dihydroartemisinin-piperaquine; PQ, primaquine.

Three patients had a severe adverse event during the first year of follow-up, none of which seemed to be related to the study drugs or malaria infection. One patient developed pericarditis 10 days after treatment with DHP + PQ. The malaria slide was negative at the time of this event. Primaquine was discontinued, and the patient made a full recovery. Two patients treated with AAQ + PQ died during the 1-year follow-up period, unrelated to malaria or study drugs. A 50-year-old diabetic male patient died 9 months after treatment after an acute myocardial infarction. A 50-year-old man died 7 months after treatment; his cause of death was unknown but followed hemoptysis in the days prior to death.

## DISCUSSION

The recent guideline of the Indonesian Ministry of Health for treatment of uncomplicated vivax malaria includes 2 first-line ACTs, AAQ and DHP [10]. We compared the efficacy and safety of these combinations in radical treatment regimens with PQ in the normal context of use (ie, without G6PD testing). In the setting of North Sumatera, both treatment regimens were safe and efficacious for cure of the blood-stage infection. Hemolysis after treatment with PQ (0.25 mg/kg for 14 days), not requiring transfusion, was a rare event. This was because the prevalence of G6PD deficiency was relatively low (<5%) by comparison with other areas of the tropics, and the prevalent genotypes were not associated with severe deficiency.

A study from Thailand found a similar low risk for hemolysis after treatment with PQ in the same dosing scheme, without prior G6PD testing [13]. The Mahidol variant (487G→A) is also the most common G6PD variant in the western part of Thailand.

We screened patients for G6PD deficiency at the end of follow-up with a fluorescent spot test. This identified another 7 patients who were G6PD deficient according to this test, of whom 1 male was hemizygous for the Mahidol variant and another male showed the relatively common 1311C→T intron 11 nt93T→C mutation, both associated with mild G6PD deficiency [14, 15]. In total, 3.3% of patients had a variant G6PD genotype, which compares to an earlier study in North Sumatera showing a 5% prevalence of G6PD deficiency [5]; the slightly lower prevalence in vivax patients in the current study might relate to the protective effect of G6PD deficiency against malaria [16–18]. A total of 4 of 9 (44%) patients with a positive fluorescent screening test denoting G6PD deficiency had a normal G6PD genotype, indicating suboptimal specificity of the test, which could be related to the presence of additional sources of oxidative stress (eg, deriving from food or drugs) not accounted for in the test. Only 5 of 331 (1.5%) patients developed significant intravascular hemolysis (>2 g/dL hemoglobin drop), none of whom required a blood transfusion. Another 3 of 331 (0.9%) had methemoglobin levels >20% related to PQ treatment, without any other clinical signs. Most (7 of 8 [87.5%]) adverse events occurred within the first 7 days of treatment and all quickly resolved. Our findings suggest that both regimens including low-dose PQ can be deployed safely in this setting of low prevalence and “mild-type” G6PD deficiency, provided that the risks are acknowledged and that adequate follow-up can be assured. It should be noted that PQ is contraindicated during pregnancy. Implementation of G6PD testing should be a priority in *P. vivax* endemic settings, but where this is currently not feasible, a suggested follow-up scheme is a daily visit during the first 7 days of treatment with hematocrit or hemoglobin levels measured at diagnosis and 3 and 7 days after start of treatment. If hemoglobinuria occurs, then PQ should be stopped. Simple color cards to aid detection of hemoglobinuria may be useful.

Both treatments resulted in a rapid clinical and parasitological cure, fast gametocyte clearance, and good therapeutic efficacy at 42 days. Only 1 patient treated with DHP + PQ had early treatment failure. In vivax malaria, genotyping cannot distinguish between relapse and reinfection, as more than half of the relapse infections in endemic areas are caused by reactivation of liver schizonts with a different genotype [19]. Because the natural history of relapse infections in North Sumatera is not known and this study did not include a control arm without PQ administration, we cannot assess with certainty the efficacy of this low-dose PQ regimen for preventing relapse infection. In our study, 28 of 289 (9.7%) patients had recurrent infections after 1 year of follow-up. In comparison, in patients returning from highly endemic Papua Indonesia to nonendemic Java, relapse rates were comparable, with 2 of 36 (6%) relapses after treatment with DHP + PQ combined with a higher dose (30 mg) of PQ [20]. However, hypnozoite sensitivity may vary geographically. In our study, the ratio between *P. falciparum* and *P. vivax* infections was 6.5:1 during screening and 2:1 during follow-up, suggesting that a proportion of the late recurrent infections were relapse infections. Efficacy trials of ACT regimens with and without PQ are now being planned and implemented throughout Asia to assess the dose-dependent relapse-preventing efficacy of PQ in the treatment of vivax malaria.

Both relapse and recurrent infections are suppressed by the posttreatment prophylactic effect of the long half-life partner drug in the ACT used for treatment. The terminal half-life of the active metabolite of amodiaquine, desethylamodiaquine, is approximately 21 days [21], compared to 28–35 days for piperaquine [22]. In our study the earliest recurrence with AAQ + PQ was indeed earlier (at 54 days) than with DHP + PQ (at 83 days), but with longer follow-up this advantage disappeared. After 1 year, the time to recurrent infection was no longer statistically different between treatment groups.

Both regimens used in this study were well tolerated, although DHP + PQ was associated with significantly fewer (mild) adverse events than AAQ + PQ, as has also been reported in other studies [23, 24]. In addition to its longer posttreatment prophylactic effect, this makes DHP + PQ an attractive alternative to AAQ + PQ for the treatment of uncomplicated vivax malaria, and could be a further step to harmonization of the treatment of falciparum and vivax malaria in Indonesia.

This study has several limitations: 12% of patients were lost for follow-up at day 42, related to poor accessibility of some areas in rural northern Sumatera, and 22% were not tested for G6PD status at the end of the study, so our prevalence estimate may be imprecise. Patients with hemolysis were not formally assessed for changes in renal function, but no patient reported anuria or developed symptoms of renal failure during follow-up.

The number of G6PD-deficient patients in the current study was low, and because enzyme activity can vary considerably even within specific genotypes, assessment of the hemolysis risk after low-dose PQ within specific genotypes requires larger studies. Further prevalence studies on the genetic variants of G6PD and their corresponding phenotypes in various parts of Indonesia will be required to generalize our current findings to other parts of Indonesia.

In conclusion, radical treatment with AAQ or DHP, both combined with low-dose PQ (0.25 mg/kg for 14 days), without prior testing for G6PD deficiency proved a safe and efficacious treatment for uncomplicated *P. vivax* in North Sumatera. DHP + PQ was better tolerated and had a longer posttherapeutic prophylactic effect.
